# Effectiveness of an online self-management tool, OPERAS (an On-demand Program to EmpoweR Active Self-management), for people with rheumatoid arthritis: a research protocol

**DOI:** 10.1186/s13063-019-3851-0

**Published:** 2019-12-11

**Authors:** Johnathan Tam, Diane Lacaille, Teresa Liu-Ambrose, Chris Shaw, Hui Xie, Catherine L. Backman, John M. Esdaile, Kimberly Miller, Robert Petrella, Linda C. Li

**Affiliations:** 1 Arthritis Research Canada, Milan Ilich Arthritis Research Centre, 5591 No. 3 Road, Richmond, BC V6X 2C7 Canada; 2 Department of Medicine, University of British Columbia, 2775 Laurel Street, 10th Floor, Vancouver, BC V5Z 1M9 Canada; 3 Department of Physical Therapy, University of British Columbia, Friedman Building, 212 – 2177 Wesbrook Mall, Vancouver, BC V6T 1Z3 Canada; 4 School of Interactive Arts and Technology, Simon Fraser University, 250-13450 102 Avenue, Surrey, BC V3T 0A3 Canada; 5 Faculty of Health Sciences, Simon Fraser University, 8888 University Drive, Burnaby, BC V5A 1S6 Canada; 6 Department of Occupational Science & Occupational Therapy, University of British Columbia, 325-2211 Wesbrook Mall, Vancouver, BC V6T 2B5 Canada; 7 Sunny Hill Health Centre for Children, BC Children’s Hospital, 3644 Slocan Street, Vancouver, BC V5M 3H4 Canada; 8 Department of Family Medicine, 3rd Floor David Strangway Building, 5950 University Boulevard, Vancouver, BC V6T 1Z3 Canada

**Keywords:** Rheumatoid arthritis, e-health, Self-monitoring, Physical activity, Self-management

## Abstract

**Background:**

Active self-management is a process where patients are fully engaged in managing their health in daily life by having access to contextualized health data and tailored guidance to support a healthy lifestyle. This study aims to determine whether an e-health intervention that incorporates symptom/disease activity monitoring and physical activity counselling can improve self-management ability in patients with rheumatoid arthritis (RA).

**Methods:**

The ‘Empowering active self-management of arthritis: Raising the bar with OPERAS (an On-demand Program to EmpoweR Active Self-management)’ project is a randomized controlled trial that uses a delayed control design. One hundred thirty-four participants with RA will be randomly assigned to start the intervention either immediately (immediate group) or 6 months later (delayed group). The intervention involves (1) use of a Fitbit-compatible web app to record and monitor their RA disease activity, symptoms, and time spent on physical activity and a Fitbit; (2) group education and individual counselling by a physiotherapist (PT); and (3) six phone calls with a PT. The primary outcome measure is self-management ability measured by the Patient Activation Measure. Secondary outcome measures include disease status, fatigue, pain, depressive symptoms, and characteristics of habitual behavior and also time spent in physical activity and sedentary activity with a wearable multi-sensor device (SenseWear Mini). After the 6-month intervention, we will interview a sample of participants to examine their experiences with the intervention.

**Discussion:**

The results of this study will help to determine whether this technology-enhanced self-management intervention improves self-management ability and health outcomes for people living with RA. A limitation of this study is that participants will need to self-report their symptoms, disease status, and treatment use through questionnaires on the OPERAS web app. The user-friendly interface, reminder emails from the research staff, and tailored guidance from PTs will encourage participants to actively engage with the app.

**Trial registration:**

Date of last update in ClinicalTrials.gov: January 2, 2019. ClinicalTrials.gov Identifier: NCT03404245.

## Background

Self-management is a key component of successful chronic disease management [1].

Broadly, it is a process where patients are actively participating in a variety of activities that contribute to lessening of the physical and emotional impact of their illness. Such activities include adhering to their treatment plan, being physically active, and seeking medical help when the treatment target is not met. It is important to make self-management a priority as patients can benefit from learning about how their daily activities and treatments correlate with their symptoms and health status on an ongoing basis. These contextualized health data provide a baseline for patients to determine whether their symptoms warrant seeking medical attention and could inform their decisions about daily activities. For example, a patient who is aware of how much pain and fatigue they experience after a day of housework will know to pace their activities the next day.

Chronic disease self-management is hard work; it can be difficult to follow treatment regimes in the context of busy lives and fluctuating symptoms. There is a need to develop a multifaceted approach that can promote self-management and adherence to recommended treatments. As an example, for people with rheumatoid arthritis (RA), there is ample evidence to support early and persistent use of disease-modifying anti-rheumatic drugs (DMARDs) [2]. However, for people who started RA medications, the adherence rates are as low as 30% [3]. Furthermore, despite the compelling evidence supporting a physically active lifestyle in reducing symptoms [4–7], the majority of patients do not meet the minimum recommended level of moderate to vigorous physical activity (MVPA) [8].

In general, patients feel a moral obligation to “manage well” [9]. However, some become disengaged from self-management activities [10] because of a frustration from managing their health on a trial-and-error basis [11]. The current practice relies on patients applying their self-management knowledge and skills in daily life with little feedback on their performance or how their actions affect their health. In light of these findings, we believe that there is a need for a multifaceted approach that provides support in terms of knowledge, skill development, and timely advice from health professionals as well as motivational support for patients to be engaged in their care and to stay physically active.

### OPERAS web application

The OPERAS (On-demand Program to EmpoweR Active Self-management) web app was designed to engage patients in monitoring and managing their health. Building from two existing software programs, the Arthritis Health Journal (AHJ) [12] and FitViz [13], OPERAS allows users to track symptoms, self-management goals, medication use, and physical activity. The last of these is automatically measured by Fitbit, Inc. San Francisco, CA. Using OPERAS, a physiotherapist (PT) will provide remote counselling to guide the user in setting realistic physical activity goals. Based on the users’ goals, the PT can adjust parameters of physical activity intensity and duration to provide automated personalized feedback on their goal attainment.

The objectives of this study are to (1) assess the efficacy of OPERAS at improving self-management ability of people with RA in a 6-month randomized controlled trial (RCT); (2) explore the effect of OPERAS on disease status, pain, fatigue, depressive symptoms, and characteristics of habitual behavior; and (3) assess barriers to implementation and sustainability of OPERAS from the perspectives of participants involved in the study.

## Methods/design

### Study design

**Fig. 1:**
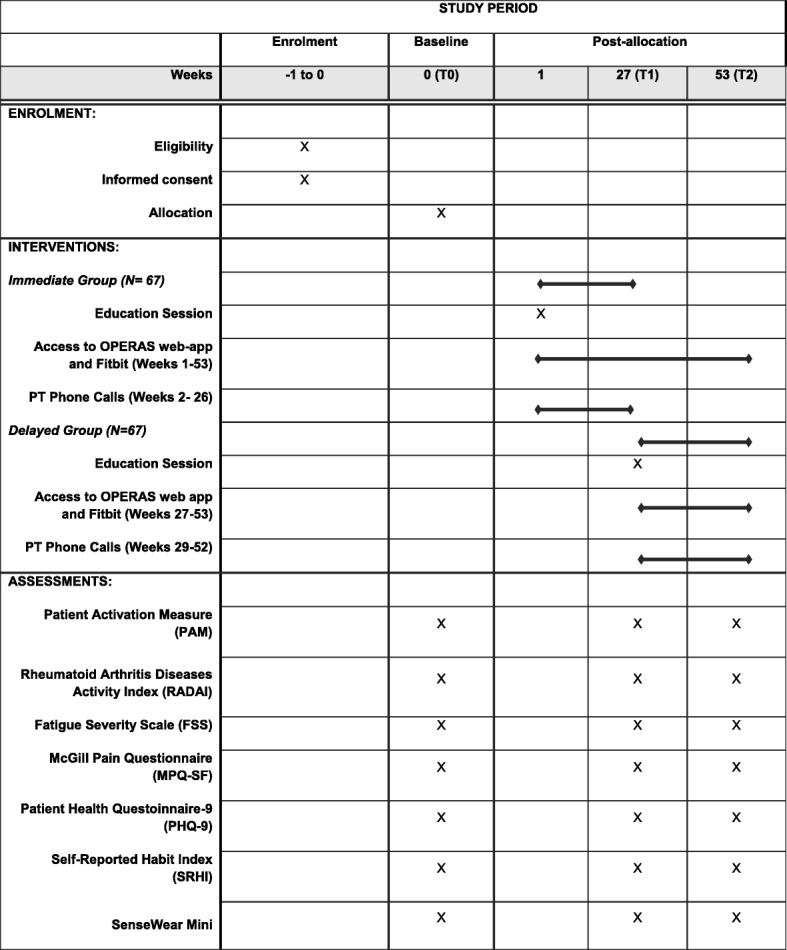
Standard Protocol Items: Recommendation for Interventional Trials (SPIRIT) figure

The ‘Empowering active self-management of arthritis: Raising the bar with OPERAS (an On-demand Program to EmpoweR Active Self-management)’ project will use a mix of quantitative and qualitative research methods. The RCT will employ a delayed control design whereby participants will be randomly assigned to start the 6-month intervention immediately (immediate intervention [II] group) or 6 months later (delayed intervention [DI] group) by using a 1:1 allocation ratio. Participants will be assessed three times throughout the study (Fig. 1, Additional file 1). At the end of the intervention, we will invite a sample of participants to be interviewed regarding their experiences.

### Participants

Individuals are eligible for the study if they have (1) a rheumatologist-confirmed diagnosis of RA, (2) no joint surgery in the past 6 months, (3) no history of acute injury to any joints in the past 6 months, (4) an email address and daily access to a computer or mobile device, (5) the ability to speak and understand English, and (6) not previously participated in studies involving the AHJ or physical activity counselling. We will exclude people who should not be physically active without medical supervision, as identified by the Physical Activity Readiness Questionnaire (PAR-Q) [14]. If participants fail the PAR-Q, a physician’s note will be required to determine whether they are eligible.

Participants will be recruited from rheumatology clinics in Metro Vancouver and through the network of patient groups, including Arthritis Consumer Experts and Arthritis Research Canada’s Arthritis Patient Advisory Board. We will also post study information on social media (Facebook, Twitter, Kajiji, and Craigslist) and Arthritis Research Canada’s website.

Once participants complete the screening process and are deemed fully eligible for the study, the study coordinator will provide detailed information on the study, and a consent form will be emailed to the participant. When a signed consent form is obtained (e-signature or scanned copy), the participant will be enrolled. All potential and consented participants will be assigned a study ID that links to their personal and contact information. This information will be stored in a password-protected file on a secure database accessible to the research team only.

### The intervention

Participants randomly assigned to the II group will receive the 6-month intervention immediately. They will attend a 2-h in-person session where they will (1) participate in a group education session from a study PT about self-management and physical activity for people with RA, (2) receive a Fitbit Inspire and an orientation to the OPERAS web app, and (3) receive individual counselling from a PT trained in motivational interviewing [15]. All study PTs will follow the Brief Action Planning approach [16], whereby participants will be guided to set their own physical activity goals, develop an action plan, and identify barriers and solutions.

We will ask participants to wear the Fitbit throughout the intervention period and use the OPERAS app to record their RA disease activity, symptoms, treatment use, and self-management action plans. If participants experience any discomfort wearing the Fitbit, we will advise them to stop wearing it but continue using the OPERAS web app. Disease activity will be tabulated from participants’ self-reported measures, including the Rheumatoid Arthritis Disease Activity Index (RADAI), Visual Analogue Scale (VAS), and the Health Assessment Questionnaire (HAQ). Participants will record their symptoms twice a week during periods of more active disease (i.e., a disease activity score above 4) and once every 2 weeks during periods of stable disease (i.e., no sudden increases in disease activity score). They will also be asked to log on and review their physical activity goal achievements at least once a week. Participants who have not recorded their disease activity for more than 2 weeks will receive an email reminder from the research team.

II participants will receive a phone call from their PTs at weeks 2, 4, 6, 8, 13, and 26 to review their physical activity goals and accomplishments. PTs will coach participants to modify their goals if they are ready.

The DI group will receive the same intervention in week 27. Participants will receive a monthly newsletter of arthritis news, which is unrelated to disease management prior to the intervention period.

After the 6-month intervention period, both II and DI participants can keep the Fitbit and their OPERAS app account but will not have access to a PT. There is no requirement for participants to stop their medical treatments and non-pharmacological care during their participation in the study.

### Measurements

All participants will be assessed three times throughout the study: at baseline (T0), week 27 (T1), and week 53 (T2). Baseline measurements will be completed before randomization.

Research personnel performing data processing and analysis will be blinded to the group assignment. We will not be blinding the group assignment to the study coordinator, who will be facilitating delivery of the intervention, nor the participants who will be receiving it.

### Outcomes

#### Primary outcome

Self-management ability will be assessed through the Patient Activation Measure (PAM), a 13-item self-reported measure of individual’s confidence in managing chronic diseases [17, 18]. Each item has a 4-point response (1 = ‘strongly disagree’ and 4 = ‘strongly agree’), and the aggregate raw score is converted to 0–100 [15]. Hibbard et al. [17] described a four-stage activation model based on the standardized scores of PAM: (1) believing an active role is important (PAM score <47), (2) having confidence and knowledge to take action (47.1–55.1), (3) taking action (55.2–67), and (4) maintaining healthy behaviors despite setbacks (>67.1). PAM has demonstrated internal consistency (Chronbach’s α >0.85) [17] and construct validity with health status measures (e.g., 36-Item Short Form Survey [19]) and healthy behaviors such as exercise, healthy eating, and medication adherence [19].

#### Secondary outcomes

We will assess disease status through the RADAI, which categorizes disease activity into remission, low, moderate, and high states [20]. RADAI has five components: (1) global disease activity, (2) joint tenderness/swelling, (3) pain, (4) morning stiffness, and (5) number/severity of painful joints. Its validity was supported by moderate correlations with physicians’ assessment of disease activity (*r* = 0.54), swollen joint count (*r* = 0.54), and C-reactive protein value (*r* = 0.43) [20].

Pain will be measured with the McGill Pain Questionnaire (MPQ-SF), which consists of 15 pain-related words that can be rated from 0 to 3, with the higher number being more severe [21]. We will measure fatigue by using the Fatigue Severity Scale (FSS), a nine-item questionnaire that has demonstrated internal consistency (Cronbach’s α = 0.89) [22]. The FSS is also moderately correlated with pain (*r* = 0.68) and depression (*r* = 0.46) [22, 23]. We will also assess participants’ mood by using the Patient Health Questionnaire-9 (PHQ-9) [24], which consists of nine questions that correspond to the diagnostic criteria for major depressive disorder. A total score of greater than 11 indicates the presence of major depressive disorder [24].

The Self-Reported Habit Index (SRHI) will be used to measure characteristics of habitual behavior. It is a 12-item scale, rated on a 7-point Likert scale, with higher scores indicating a stronger habit or behavior that is done frequently, automatically, and without thinking about it [25, 26]. Participants will rate their strength of habit for three specific behaviors: sitting during leisure time at home, sitting during usual occupational activities, and walking outside for 10 min or more.

We will measure participants’ time spent in physical activity and sedentary activity with SenseWear Mini, BodyMedia, Inc., Pittsburgh, PA. It integrates tri-axial accelerometer data, physiological sensor data, and personal demographic information to estimate steps, energy expenditure, and metabolic equivalent of tasks (METs). Tierney et al. [27] showed that the SenseWear is an appropriate tool in estimating energy expenditure during activities of daily living in people with arthritis (intraclass correlation coefficient (ICC) = 0.72). Participants will wear the SenseWear monitor over their triceps on the non-dominant arm for 7 days. Almeida et al. [28] recommended that a minimum of 4 days is required to reliably assess energy expenditure from different levels of physical activity in people with RA (ICC >0.80).

We will calculate the mean time spent in bouted MVPA per day. A bout is defined as more than 10 consecutive minutes at the level of more than 3 METs (i.e., the lower bound of MVPA) with allowance for interruption of up to one minute below the threshold [29]. We will also calculate the mean daily time spent in sedentary behaviors, with an energy expenditure of  1.5 METs or lower, occurring in bouts of 20 minutes or more during waking hours [30, 31].

### Implementation assessment

At the end of the 6-month intervention, we will conduct 1-hour interviews with a purposive sample of participants to include both men and women who have different durations of experience in using health-related apps (novice: <2 years; avid >2 years). The interview guide will explore three topics: (1) the participants’ experiences with the intervention, probing to understand their views of each aspect of the program; (2) barriers and facilitators to using OPERAS; and (3) the nature of activities they engaged in with the program.

The OPERAS app will collect program usage data of all participants and these data will be securely stored on the Arthritis Research Canada server. Information collected include (1) participants’ frequency of using the OPERAS app, (2) duration of each use, (3) adherence to using the Fitbit, and (4) adherence to the PT counselling session. The app is designed to allow researchers to obtain usage data without access to the personal health information, protecting participants’ health information privacy.

### Adverse events and data monitoring

In the follow-up questionnaires at months 6 and 9, participants will be asked to note any serious adverse events, including falls and cardiovascular/musculoskeletal events [32]. Adverse events will be reviewed by the Data and Safety Monitoring Committee, which will recommend terminating the study, if warranted. Additionally, at any time during the study period, participants will report any serious adverse events directly to the study coordinator. In the event of a severe adverse event due to participation in the study, participants will be recommended to follow-up with their family doctor as well as have the option to meet with a rheumatologist (DL) or a PT (LCL).

The Data and Safety Monitoring Committee will also be in charge of auditing trial conduct, reviewing trial processes and documents to ensure that research activities comply with the requirements of the protocol. It will review screening and consent documentation to ensure ethical trial conduct. Committee members will meet, in person or via teleconference, quarterly or when 25%, 50%, and 75% of the participants are enrolled, whichever is sooner.

### Sample size calculation

Our primary outcome measure is self-management ability as measured by the PAM [17, 18]. Turner et al. reported that patients with chronic diseases who completed a 7-week self-management program would have their mean PAM score improve from 52.2 to 60.2 at 6 months (standardized effect size = 0.65) [33]. Based on a difference of 8 points, an estimated standard deviation of 12.4, and a two-tailed analysis of variance (ANOVA), a total of 102 participants (51 per group) would be needed (90% power and α level of 0.05). We plan to recruit a total sample size of 134 (67 participants in each group) to allow for an attrition rate of about 24%.

For the implementation assessment, we will conduct qualitative interviews with 7–10 participants in each of the four purposive sampling strata, namely gender (2) and experience in using health-related apps (2). Hence, 28–40 people will be interviewed.

### Data analysis

The data collected will be de-identified and password-protected. All research personnel involved with data analysis will have access to the data files. The study coordinator will oversee the data-sharing process.

### Efficacy analyses

The intervention efficacy will follow an intention-to-treat approach. We will analyze the outcomes measured at weeks 26 and 52 in the II and DI groups. We will employ generalized linear mixed-effect models (GLMMs), adjusting for baseline value on the outcome variable and sex as covariates. These models are the most efficient and recommended statistical methods for analyzing longitudinal clinical trial data with missing data [34, 35] and can account for data missing at random without the need to perform explicit imputations of the missing values [36]. Binary indicators of group and months since baseline will allow us to test their interaction (*P* <0.05 for two-tailed Wald test), which addresses whether the outcome post-intervention is significantly better in the II group than in the DI group. Data transformation, if needed, will be applied to continuous outcomes to satisfy the assumption of normality. The sandwich estimators for GLMMs [37] will be used to compute empirical standard errors that are robust to model specifications and distributional assumptions. An unstructured variance-covariance matrix will be used to model the within-subject error variance-covariance for continuous outcomes. The quality of statistical inferences will be substantiated through rigorous model checking and validation techniques.

### Implementation analysis

For the qualitative interviews, we will conduct an iterative content analysis, whereby codes will be identified and revised as interviews are analyzed. Initial open coding will be followed by clustering the labels into thematic categories [38]. Quotes representative of the thematic categories will be identified to illustrate experiences of participants with the intervention as well as barriers and facilitators in meeting their physical activity goals. If distinct themes based on gender or age are apparent, data will be reanalyzed within subgroups. Descriptive analysis will be conducted for all participants’ usage data from the OPERAS app.

An iterative content analysis will be used to identify and revise codes in the qualitative interviews. We will start with an initial open coding and then cluster the labels into thematic categories [38]. Quotes representative of the thematic categories will be identified to illustrate experiences of participants in using OPERAS as well as barriers and facilitators in using the program. We will reanalyze the data within subgroups if there are distinct themes based on gender or age. We will also conduct a descriptive analysis for all participants’ usage data.

### Publication policy

The principal and co-investigators will review all potential publications resulting from the data collected. Trial results, full study report, and study protocol will be made available to all participants, physicians, patients, participating PTs, and the general public. There are no plans to grant public access to a de-identified data set.

## Discussion

### Potential impact and significance

For patients, active self-management is a fundamental but often neglected component in current models of care [39]. To this end, this study will evaluate the effectiveness of an e-health intervention at empowering patients to engage in self-care activities, such as monitoring their health and being physically active. Should the intervention prove to be effective for patients with RA, we believe OPERAS has the potential to be adaptable for patients with other chronic diseases to address the issue of self-management.

### Limitations of the study

A challenge of OPERAS is that it requires participants to actively record their symptoms/disease status and treatment use, although physical activity tracking will be achieved automatically. To encourage participants to record data, OPERAS is designed with a user-friendly interface to enter the information, and we have a built-in protocol to send reminders after a period of non-use. Based on feedback from our patient partners, we included the self-reported component because it will enable the program to provide a personalized picture of individuals’ health in relation to their activities and treatment use.

Another limitation of OPERAS is that participants need to wear and use a Fitbit continuously over the 6-month intervention. To minimize non-use, we selected the Fitbit Inspire, which can be worn on the wrist 24 h a day. In a previous study involving the Fitbit Flex, patients with arthritis were able to wear and use the device over an extended period of time [40]. To ensure that the Fitbit is being used properly, the research coordinator will monitor the physical activity data synchronization with the app. Since the Fitbit is a commercial product, it is possible that participants will acquire one during the non-intervention period. To encourage adherence to the protocol, we will inform participants that they will receive a Fitbit from us free of charge.

### Trial status

We obtained ethics approval for this study on April 17, 2018. The study has also been registered on ClinicalTrials.gov, and we began recruitment in January 2019. We expect to complete recruitment in December 2020.

## Supplementary information

**Additional file 1.** Standard Protocol Items: Recommendation for Interventional Trials (SPIRIT) 2013 Checklist: Recommended items to address in a clinical trial protocol and related documents*.

## Data Availability

Not applicable.

## Abbreviations

AHJ: Arthritis Health Journal
DI: Delayed intervention
FSS: Fatigue Severity Scale
GLMM: Generalized linear mixed-effect model
ICC: Intraclass correlation coefficient
II: Immediate intervention
MET: Metabolic equivalent of task
MVPA: Moderate to vigorous physical activity
OPERAS: On-demand Program to EmpoweR Active Self-management
PAM: Patient Activation Measure
PAR-Q: Physical Activity Readiness Questionnaire
PT: Physiotherapist
RA: Rheumatoid arthritis
RADAI: Rheumatoid Arthritis Disease Activity Index
RCT: Randomized controlled trial
